# Single‐cell sequencing analysis reveals cetuximab resistance mechanism and salvage strategy in colorectal cancer

**DOI:** 10.1002/ctm2.70151

**Published:** 2024-12-31

**Authors:** Shiyun Chen, Zhaoli Tan, Xiaojie Wu, Yanli Lin, Xiang Li, Yumeng Cui, Weiling Man, Fang Pang, Yanghua Li, Faliang Shi, Lu Han, Miaomiao Gou, Li Zhou, Zhikuan Wang, Youliang Wang, Guanghai Dai

**Affiliations:** ^1^ Department of Oncology the Fifth Medical Center Chinese PLA General Hospital Beijing China; ^2^ Laboratory of Advanced Biotechnology Beijing Institute of Biotechnology Beijing China; ^3^ Department of Sequencing Analysis Beijing Easyresearch Technology Limited Company Beijing China

Dear Editor,

1

Through single‐cell sequencing analysis, we identified heterogeneity and evolutionary trends in tumour cell subsets, as well as key signalling pathways involved in acquired resistance to cetuximab. Our findings suggest that celecoxib, which simultaneously inhibits ERK and SMAD signalling pathways, could effectively counteract cetuximab resistance in treating colorectal cancer (CRC).

Cetuximab is the preferred treatment for *RAS/RAF* wild‐type metastatic CRC.^1^ However, its efficacy is significantly curtailed by the development of resistance, with up to 70% of patients developing resistance within one year of cetuximab therapy.^2^ Our previous investigation highlighted that *PRSS* is intricately linked to cetuximab resistance.^3^ Furthermore, several key genes implicated in EGFR monoclonal antibody resistance have been identified, including *EGFR*,^4^ *KRAS*,^5^ *PIK3CA* ^6^ and *MET*.^7^ Despite these findings, reversing cetuximab resistance remains challenging. Although no large‐scale clinical research has confirmed that celecoxib reverses cetuximab resistance, it is deemed a promising candidate for combination therapy in light of its extensive antitumor efficacy across a spectrum of cancers and its prevalent application in clinical practice.^8^

In this study, we systematically investigated the molecular mechanisms and evolutionary pathways of acquired resistance via single‐cell RNA sequencing (scRNA‐seq) analysis. Initially, we established two independent cetuximab resistance models in CRC cell lines DiFi and LIM1215 (Figure 1A,B and Figure S1). Utilizing scRNA‐seq, underlying mechanisms of cetuximab resistance were explored. Our findings revealed that CRC cells generated multiple subsets of tumour cells during the transition period (DiFi‐R‐T and LIM‐R‐T) of acquired drug resistance (Figure 1C and Figure S2A). Each cellular subset exhibited distinct highly expressed marker genes (Figures S2B and S3).

**FIGURE 1:**
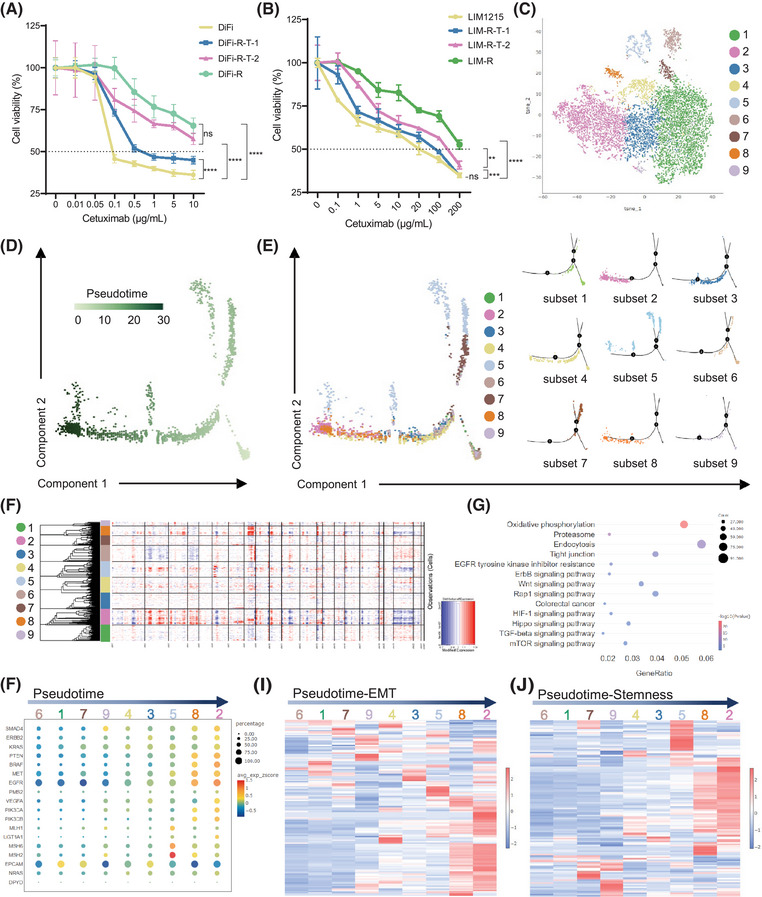
Single‐cell sequencing analysis reveals cellular evolutionary trajectories during acquired resistance to cetuximab. (A) DiFi cell lines were tested for cell sensitivity to cetuximab at various stages during induction of resistance (DiFi‐R‐T‐1, induced for 2 months; DiFi‐R‐T‐2, induced for 4 months; DiFi‐R, induced for more than 6 months). (B) Sensitivity testing of cells to cetuximab at various stages of LIM1215‐induced resistance (LIM‐R‐T‐1, induced for 2 months; LIM‐R‐T‐2, induced for 4 months; LIM‐R, induced for more than 6 months). (C) t‐SNE visualization of multiple cell subsets within DiFi‐R‐T. (D) Pseudotime analysis coloured by simulated time within DiFi‐R‐T. (E) Pseudotime analysis coloured by cell subset within DiFi‐R‐T. (F) Inferred copy number variation (inferCNV) of each cell subset within DiFi‐R‐T. (G) Kyoto Encyclopedia of Genes and Genomes (KEGG) analysis of differentially expressed genes (DEGs) (Link to Figure S5) within DiFi‐R‐T. (H) Bubble plots of colorectal cancer treatment‐related gene expression following pseudotime within DiFi‐R‐T. (I) Heat map of epithelial‐mesenchymal transition (EMT) gene set expression within DiFi‐R‐T. (J) Heat map of the expression of the tumour stemness gene set within DiFi‐R‐T. *p < .05, **p < .01, ***p < .001 and ****p < .0001.

A pseudotime analysis using DiFi‐R‐T was conducted to investigate the evolutionary trajectory of cell subsets. The results indicated that the trajectory of cell subset evolution followed a path from initial subsets such as subset 6 and subset 1 to terminal subsets like subset 2 (Figure 1D,E). Similarly, a pseudotime analysis with LIM‐R‐T demonstrated a transition from initial cell subsets like subset 1 and subset 7 to terminal subset 8 (Figure S4). Copy number variation (CNV) analyses revealed significant CNV disparities in the terminal subsets of both cell lines compared to other subsets, suggesting that terminal subsets possess a higher degree of malignancy relative to other subsets. These terminal subsets likely represent the group of cells that successfully transitioned into a fully cetuximab‐resistant subset during resistance induction (Figure 1F and Figure S2C).

Based on this foundation, differential gene analysis (Figures S2D and S5) and KEGG enrichment analysis were conducted on the terminal subset and all other cellular subsets. This revealed several signalling pathways potentially associated with drug resistance, including EGFR and TGF‐β/SMAD signalling pathways (Figure 1G and Figure S2E). Subsequently, a pseudotime framework was established to examine the evolution of genes closely related to CRC treatment in current clinical practice. The findings indicated that, over pseudotime, genes pertinent to intestinal cancer clinical treatment, such as *KRAS*, *NRAS* and *BRAF*, exhibited a gradual increase in the proportion of upregulation (Figure 1H and Figure S6A and S7A,B).

Previous studies have revealed a strong association between cancer cell stemness (CSCs), epithelial‐mesenchymal transition (EMT) and drug resistance in various tumours.^9^ In light of this, we analyzed the EMT and CSCs gene set scores during the development of acquired resistance to cetuximab. Our analysis revealed that the resistance terminal subsets of both cell lines, DiFi‐R‐T and LIM‐R‐T, exhibited the highest percentage of positive cells and scores (Figures S6B–E and S7C,E). Furthermore, following the pseudotime trajectory, we observed a gradual increase in the upregulation percentage of EMT gene and CSCs gene expression (Figure 1I,J and Figures S6F–H and S7D,F).

To find common features in the cetuximab resistance process of two independent CRC cell lines, DiFi and LIM1215, we focused on key intersecting genes identified via scRNA‐seq analyses of DiFi‐R‐T and LIM‐R‐T (Figure S10). Protein‐protein interaction network analysis suggested that SMAD and MAPK family members might play crucial roles in driving cetuximab resistance (Figure 2A). RNA sequencing (Figure S11) on parental lines (DiFi and LIM1215) and drug‐resistant lines (DiFi‐R and LIM‐R) alongside scRNA‐seq analysis(Figures S8 and S9) of cetuximab‐resistant transition‐phase cells (DiFi‐R‐T and LIM‐R‐T) was conducted. These analyses revealed a strong correlation between the activation of the TGF‐β/SMAD and MAPK/ERK signalling pathways and acquired resistance to cetuximab. Further validation through qPCR and western blot analysis confirmed significantly elevated p‐SMAD2/3 and p‐ERK1/2 expression levels in cetuximab‐resistant cell lines (Figure 2B,C), and these elevated levels could not be inhibited by cetuximab (Figure 2D).

**FIGURE 2:**
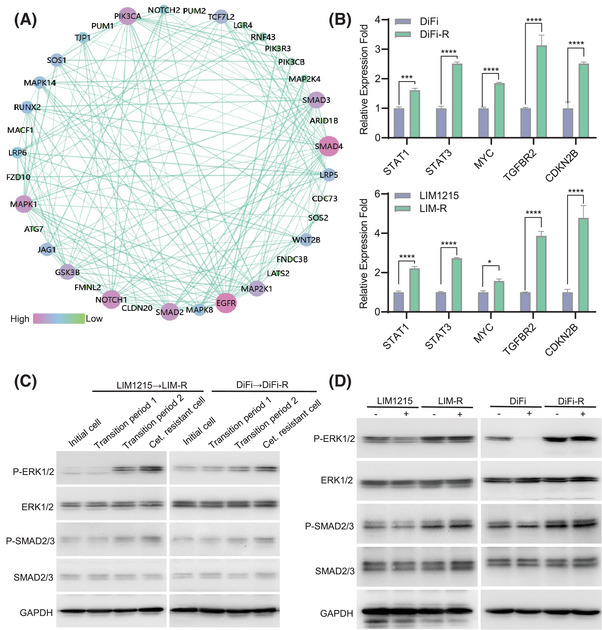
Cetuximab resistance is closely associated with the MAPK/ERK signalling pathway and SMAD signalling pathway. (A) Protein‐protein interaction (PPI) network consisting of key intersecting genes in DiFi‐R‐T and LIM‐R‐T analysis. (B) Quantitative reverse transcription polymerase chain reaction (qRT‐PCR) was performed to detect the upstream and downstream gene expression of MAPK/ERK and SMAD signalling pathways. (C) Western Blot assay of ERK and SMAD signalling pathways during cetuximab‐induced drug resistance in intestinal cancer cell lines. (D) Western Blot detection of ERK and SMAD signalling pathways after treatment of parental sensitive and resistant cell lines with cetuximab. *p < .05, ***p < .001 and ****p < .0001.

To explore the effects of ERK and SMAD pathways on cetuximab resistance, three drugs—celecoxib, SB‐431542, and U0126—were selected. Celecoxib inhibits both ERK and SMAD pathways.^10^ SB‐431542 is a TGF‐β/SMAD inhibitor and U0126 is a MAPK kinase inhibitor. Experimental results indicated that specific concentrations of these drugs could partially inhibit SMAD and ERK pathways (Figure 3A,B) without compromising cell viability (Figure 3C,D). These optimal concentrations were subsequently used in combination with cetuximab for further experiments. Notably, the combination of cetuximab and celecoxib resulted in the most significant decrease in cell viability in both DiFi‐R and LIM‐R cell replicate experiments, while U0126 and SB‐431542 enhanced resistant cells' response to cetuximab (Figure 3E,F). In groups not treated with cetuximab, there were no significant differences in proliferation (Figure 4A), apoptosis (Figure S12) and migration(Figure S13A,C) rates. However, in the presence of cetuximab, cell proliferation and migration were inhibited, and apoptosis was increased in the celecoxib, SB‐431542, and U0126 groups, with the most pronounced changes observed in the cetuximab + celecoxib group (Figure 4B–D and Figure S13B,D). Subsequent in vivo experiments demonstrated a more significant reduction in tumour growth in the cetuximab and celecoxib combination group compared to the cetuximab monotherapy group (Figure 4E and Figure S14).

**FIGURE 3:**
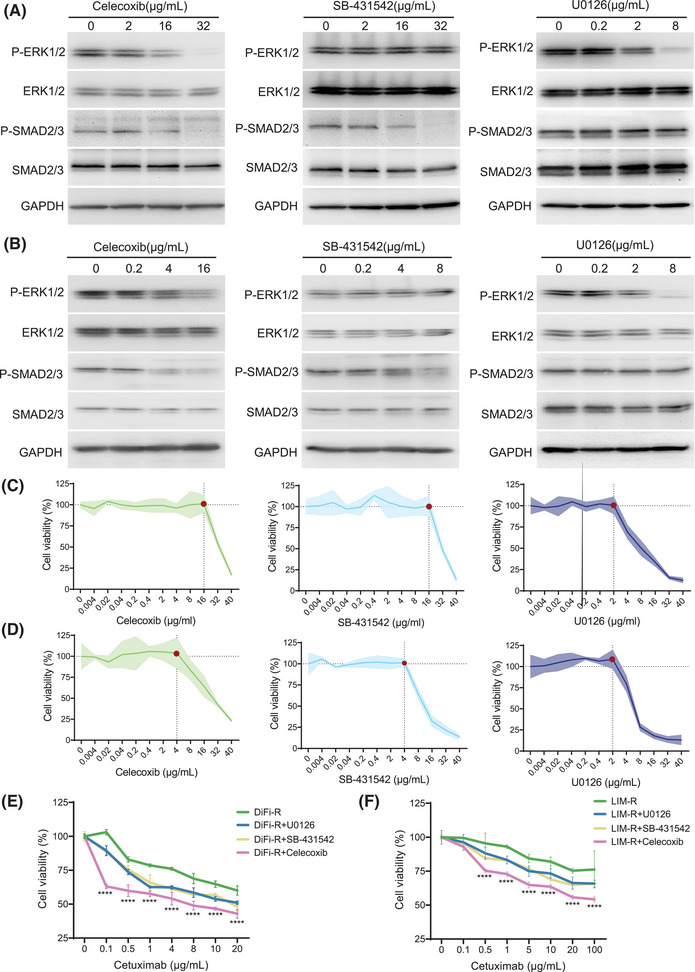
The use of celecoxib significantly restored the sensitivity of resistant lines to cetuximab. (A) Western Blot assay for inhibition of ERK signalling pathway or SMAD signalling pathway after treatment of cetuximab‐resistant line DiFi‐R with different concentrations of celecoxib, SB‐431542 and U0126 for 72 h. (B) Western Blot detection of inhibition of ERK signalling pathway or SMAD signalling pathway after 72 h of different concentrations of celecoxib, SB‐431542 and U0126 treatment of cetuximab‐resistant line LIM‐R. (C) Cell viability assay of different concentrations of celecoxib, SB‐431542 and U0126 treated cetuximab‐resistant line DiFi‐R for 72 h. (D) Cell viability assay of cetuximab‐resistant line LIM‐R treated with different concentrations of celecoxib, SB‐431542 and U0126 for 72 h. (E) Cell viability assay of resistant lines DiFi‐R after 72 h of treatment with different concentrations of cetuximab in combination with celecoxib/SB‐431542/U0126. (F) Cell viability assay of resistant lines LIM‐R after 72 h of treatment with different concentrations of cetuximab in combination with celecoxib/SB‐431542/U0126. (Red points in C and D: The optimal threshold for partially inhibiting the SMAD and ERK pathways without compromising cell viability). ****p < .0001.

**FIGURE 4:**
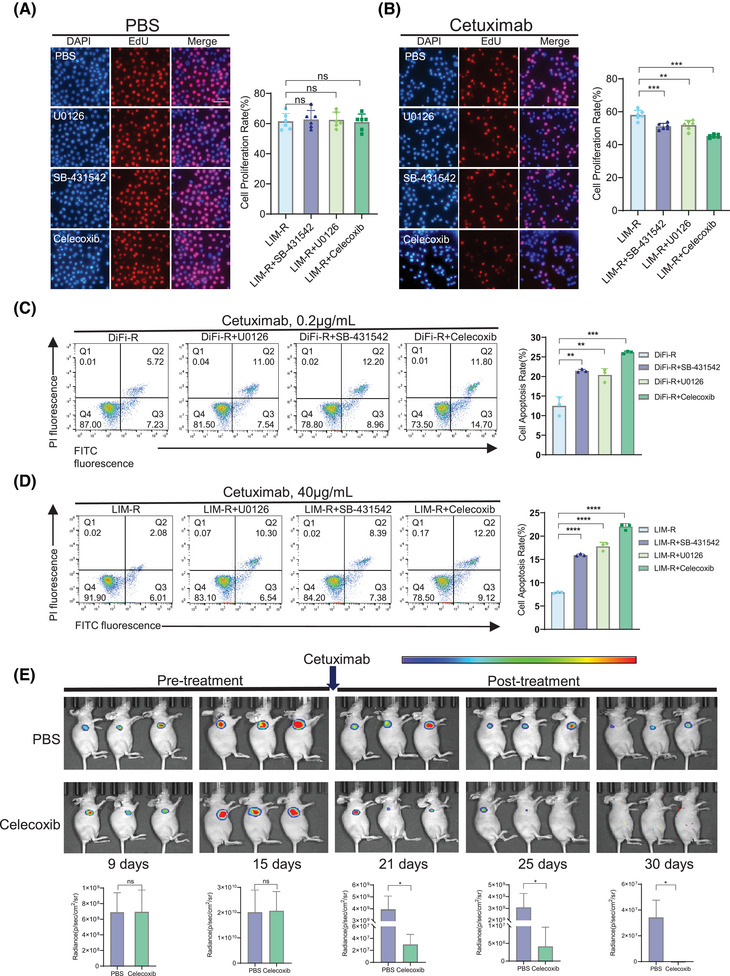
The combination of celecoxib and cetuximab synergistically inhibits proliferation and promotes apoptosis of drug‐resistant lines. (A) 5‐ethynyl‐2′‐deoxyuridine (EdU) assay for the proliferation rate of LIM‐R among the groups when not combined with cetuximab. (B) EdU assay for the proliferation rate of LIM‐R in the control, celecoxib, SB‐431542 and U0126 groups when combined with cetuximab (40 µg/mL). (C) The apoptosis rate of the parental sensitive line DiFi, the control group of the drug‐resistant line DiFi‐R, the U0126 group, the SB‐431542 group and the celecoxib group when cetuximab was present (0.2 µg/mL). (D) Apoptosis rates in the parental sensitive line LIM1215, the control group of the drug‐resistant line LIM‐R, the U0126 group, the SB‐431542 group, and the celecoxib group when cetuximab was present (40 µg/mL). (E) Luminescence images at multiple time points of orthotopic LIM‐R‐luciferase tumour‐bearing nude mice following treatment with PBS or celecoxib in combination with cetuximab, respectively. *p < .05, **p < .01 and ***p < .001.

In conclusion, CRC cells exhibit considerable heterogeneity and evolutionary trends during the development of acquired resistance to cetuximab. Critical to this process are alterations in the ERK and SMAD pathways. Therefore, the simultaneous targeting of both pathways is essential for overcoming drug resistance. This innovative strategy offers substantial potential for effectively addressing cetuximab resistance.

## AUTHOR CONTRIBUTIONS

Youliang Wang, Guanghai Dai, Zhikuan Wang and Shiyun Chen: contributed to the conception and design of the study. Youliang Wang, Shiyun Chen, Zhaoli Tan and Xiaojie Wu: performed bioinformatic data analysis and wrote the first draft of the article. Shiyun Chen, Yanli Lin, Xiang Li, Yumeng Cui, Weiling Man, Fang Pang and Yanghua Li: performed the experiments. Faliang Shi, Lu Han, Miaomiao Gou and Li Zhou: performed data analysis. Youliang Wang, Guanghai Dai, Zhikuan Wang, Shiyun Chen and Zhaoli Tan: contributed to revising the manuscript. All authors reviewed the manuscript and approved the submitted version.

## CONFLICT OF INTEREST STATEMENT

The authors declare no conflict of interest.

## FUNDING INFORMATION

This work was supported by the National Key Research and Development Program of China (2019YFA0903800 and 2022YFC3600100), the National Natural Science Foundation of China (No. 82002474 and No. 82272643) and the Natural Science Foundation of Beijing Municipal (No. 7222176).

## ETHICS STATEMENT

The animal study was approved by the Animal Ethic Review Committees of the Beijing Institute of Biotechnology (Approval number: IACUC‐DWZX‐2023‐052).

## Supporting information



Supporting Information

## Data Availability

The data that support the findings of this study are available from the corresponding author upon reasonable request.

## References

[1] Yoshino T , Arnold D , Taniguchi H , et al. Pan‐Asian adapted ESMO consensus guidelines for the management of patients with metastatic colorectal cancer: a JSMO‐ESMO initiative endorsed by CSCO, KACO, MOS, SSO and TOS. Ann Oncol. 2018;29(1):44‐70. doi:10.1093/annonc/mdx738 29155929

[2] Khan KH , Cunningham D , Werner B , et al. Longitudinal liquid biopsy and mathematical modeling of clonal evolution forecast time to treatment failure in the PROSPECT‐C phase II colorectal cancer clinical trial. Cancer Discov. 2018;8(10):1270‐1285. doi:10.1158/2159-8290.Cd-17-0891 30166348 PMC6380469

[3] Tan Z , Gao L , Wang Y , et al. PRSS contributes to cetuximab resistance in colorectal cancer. Sci Adv. 2020;6(1):eaax5576. doi:10.1126/sciadv.aax5576 31911942 PMC6938705

[4] Bardelli A , Janne PA . The road to resistance: eGFR mutation and cetuximab. Nat Med. 2012;18(2):199‐200. doi:10.1038/nm.2646 22310681

[5] Klomp JA , Klomp JE , Stalnecker CA , et al. Defining the KRAS‐ and ERK‐dependent transcriptome in KRAS‐mutant cancers. Science. 2024;384(6700):eadk0775. doi:10.1126/science.adk0775 38843331 PMC11301402

[6] Xu JM , Wang Y , Wang YL , et al. PIK3CA mutations contribute to acquired cetuximab resistance in patients with metastatic colorectal cancer. Clin Cancer Res. 2017;23(16):4602‐4616. 10.1158/1078-0432.Ccr-16-2738 28424201 PMC5559326

[7] Zhong J , Li L , Wang Z , et al. Potential resistance mechanisms revealed by targeted sequencing from lung adenocarcinoma patients with primary resistance to epidermal growth factor receptor (EGFR) tyrosine kinase inhibitors (TKIs). J Thorac Oncol. 2017;12(12):1766‐1778. doi: 10.1016/j.jtho.2017.07.032 28818608

[8] Jin MZ , Jin WL . The updated landscape of tumor microenvironment and drug repurposing. Signal Transduct Target Ther. 2020;5(1):166. doi: 10.1038/s41392-020-00280-x 32843638 PMC7447642

[9] Saha S , Pradhan N , Neha B , Mahadevappa R , Minocha S , Kumar S . Cancer plasticity: investigating the causes for this agility. Semin Cancer Biol. 2023;88:138‐156. doi: 10.1016/j.semcancer.2022.12.005 36584960

[10] Jiang S , Zhao X , Chen S , et al. Down‐regulating ERK1/2 and SMAD2/3 phosphorylation by physical barrier of celecoxib‐loaded electrospun fibrous membranes prevents tendon adhesions. Biomaterials. 2014;35(37):9920‐9929. doi: 10.1016/j.biomaterials.2014.08.028 25201739

