# Supplementary material for: Single‐cell sequencing analysis reveals cetuximab resistance mechanism and salvage strategy in colorectal cancer
Source: Clin Transl Med. 2024 Dec 31;15(1):e70151. doi: 10.1002/ctm2.70151 (PMC11686085; doi:10.1002/ctm2.70151)
Supplement: Supplementary file 1 — Supporting Information [file CTM2-15-e70151-s001.pdf]

Supporting Information

Supplementary Methods 3
scRNA-seq 3
Cell culture 4
RNA-seq 4
RNA preparation and qRT-PCR 5
Western blot 6
Cell viability assay 6
Apoptosis assay 7
EdU incorporation assay 7
Transwell assay to determine cell migration 8
Xenograft mouse experiments and in vivo studies 8
Statistical analyses 9
Figure S1 Establishment of cetuximab resistance model. 10
Figure S2 Single-cell sequencing atlas of the drug resistance transition cell line LIM-R-T. 11
Figure S3 Heatmap of Marker genes characteristically highly expressed in each cell subset within DiFi-R-T. 12
Figure S4 Distribution of the various cell subsets of LIM-R-T on the pseudotime line. 13
Figure S5 Volcano plot visualization of Differentially expressed genes (DEGs) derived by comparing subset 2 with other subsets..14
Figure S6 Following pseudotime, multiple genes associated with colorectal cancer treatment, tumor stemness and EMT were progressively up-regulated in DiFi-R-T. .15
Figure S7 Following pseudotime, multiple genes associated with colorectal cancer treatment, tumor stemness and EMT were progressively up-regulated in LIM-R-T. .17
Figure S8 GSVA analysis reveals changes in multiple tumor-related signaling pathways following pseudotime in DiFi-R-T. 19
Figure S9 GSVA analysis reveals changes in multiple tumor-related signaling pathways following pseudotime in LIM-R-T. 20

Figure S10 Evolutionary trends in key intersecting genes derived from DiFi-R-T and LIM-R-T single-cell sequencing analyses following pseudotime. 21
Figure S11 RNA-seq analysis of intestinal cancer cell lines before and after cetuximab resistance. 23
Figure S12 Apoptosis rates of the parental sensitive line, the control group of the resistant line, the U0126 group, the SB-431542 group and the celecoxib group when cetuximab was not present. 24
Figure S13 Celecoxib can synergistically inhibit the migration of drug-resistant lines when combined with cetuximab. 25
Figure S14 The combination of celecoxib and cetuximab synergistically inhibits CRC growth in vivo. 26

This supplementary material has been provided by the authors to give readers additional information about their work.

Supplementary Methods

scRNA-seq

DiFi-R-T-1 and LIM-R-T-2 were made into single-cell suspensions and sequenced using the 10×Genomics single-cell platform. The single-cell capture and downstream library construction were performed according to the Single Cell 3' Library Preparation Kit. Briefly, barcoded gel beads were wrapped around individual cells to produce a single-cell gel bead emulsion (GEM), followed by reverse transcription and cDNA amplification. The cDNA libraries were sequenced using the Illumina platform, followed by a Unique molecular identifier (UMI) count matrix processed using the R package Seurat (Version: 3.1.1). The exclusion criteria for cells in this study were gene counts less than 200 or greater than 10,000, UMIs less than 1,000, or mitochondrial gene counts >20%. After filtering according to the above criteria, the remaining cells were included in downstream analysis. Graph-based clustering and T-SNE analyses were performed on cells based on their gene expression profiles using the FindClusters function in Seurat, and the FindAllMarkers function was used to find marker genes for each cell subset. The target subsets were found by inferred CNV of the Trinity CTAT Project. FindMarkers function was used to identify differentially expressed genes (DEGs). DEGs were analyzed for Gene Ontology (GO) enrichment and KEGG pathway enrichment using R, respectively. Pseudotime analysis was performed on DiFi-R-T and LIM-R-T datasets using Monocle 3. The EMT gene set (source: https://www.gsea-msigdb.org/gsea/msigdb/human/genesets.jsp) and the CSC-like

markers gene set (source: http://stemchecker.sysbiolab.eu/) were scored using the AddModuleScore function in Seurat. Estimates of pathway activity for each cell subset were provided by GSVA. Finally, PPI network analysis was performed based on the string database (Version:11.5).

Cell culture

All cells were cultured in a 37°C, 5% CO_2 sterile humidified incubator. DiFi cells were cultured in DMEM (Gibco, USA) and LIM1215 cells were cultured in 1640 medium (Gibco, USA), while intestinal cancer cell lines were induced to be resistant to cetuximab using medium containing a fixed concentration of cetuximab, with 10% fetal bovine serum (Gibco, USA) added to all media. The highly sensitive cell line DiFi was supplemented with cetuximab 10 μg/mL and the moderately sensitive cell line LIM1215 was supplemented with cetuximab 200 μg/mL. Both cell lines were subjected to drug stress screening in culture for at least 6 months, and the cells were cryopreserved for all phases during the induction of resistance (DiFi-R-T, LIM-R-T), aiming to establish stable cetuximab-resistant CRC cell models DiFi-R and LIM-R.

RNA-seq

Total cellular RNA was extracted using the RNeasy® Kit (Qiagen, GER) and ribosomal RNA was removed using the Ribosomal rRNA Removal Kit (Vazyme, China).

Transcriptome (coding mRNA) sequencing was performed based on the Illumina HiSeq sequencing platform. The raw reads quality filtering standard is to remove sequences containing adapters, over 10% unknown nucleotides, or over 50% low quality (q value ≤10) bases. The remaining high-quality FASTQ reads were aligned to the human reference genome (hg38/GRCh38). The expression of each transcript was calculated by Cuffquant. Different mRNAs were screened in terms of fold difference and corrected significance level. GO enrichment and KEGG pathway enrichment analysis of DEGs were performed using the clusterProfiler R package.

RNA preparation and qRT-PCR

Total cellular RNA was extracted using the Ambion PureLink™ Total RNA Kit (Invitrogen, USA), followed by RNA quantification using a NanoDrop1000 spectrophotometer (NanoDrop Technologies, USA). The cDNA was prepared using the High-Capacity cDNA Reverse Transcription Kit (Invitrogen, USA). Amplification of cDNA was achieved by qPCR based on Thunderbird SYBR® qPCR Mix (Toyobo, Japan) on a CFX96 real-time PCR system (Bio-Rad, USA). Internal reference was adopted from GAPDH and performed using the $2^{-\Delta\Delta Ct}$ method to obtain relatively standardized mRNA levels.

Western blot

The total protein of the cells was extracted using RIPA lysis buffer (Thermo Scientific, USA), and then the protein concentration was measured using the Pierce BCA protein assay kit (Thermo Science, USA). Proteins were up-sampled at 25 μg per well, separated by SDS-PAGE and transferred to the PVDF membrane (Millipore, USA). The membranes were closed with 5% skimmed milk and incubated overnight with primary antibodies, which were used in this study, including SMAD2/3 antibody (#3102, Cell Signaling Technology, 1:1000), Phospho-Smad2/3 antibody (#9510, Cell Signaling Technology, 1:1000), Phospho-Erk1/2 antibody (#4370, Cell Signaling Technology,1:1000), Erk1/2 antibody (#4695, Cell Signaling Technology,1:1000), GAPDH antibody (#2118, Cell Signaling Technology, 1:2000). The secondary antibody was made using the appropriate antibody coupled to horseradish peroxidase (HRP), incubated with membranes for one hour at room temperature, and protein expression was detected by enhanced chemiluminescence (ECL) detection reagent (PerkinElmer, USA) Condition.

Cell viability assay

Medium containing the target drug concentration was added to 96-well plates, and intestinal cancer cells were collected and inoculated into 96-well plates at a concentration of 2800 cells per well, and 100 μL of CellTiter Glo reagent (Promega, USA) was added to each well 72 hours later. After mixing for 2 minutes using a shaker,

the plates were allowed to stand for 10 minutes at room temperature. The absorbance at 578 nm was measured using an enzyme marker.

Apoptosis assay

Cells were digested from Petri dishes, resuspended using PBS, and counted, and 1×10^5 cells were taken and centrifuged at $1000 \times g$ for 5 minutes, and the supernatant was discarded. Cells were resuspended using 195 μL of binding solution and 5 μL of Annexin V-FITC and 10 μL of propyl iodide solution were added according to the instructions of the Annexin V-FITC Apoptosis Detection Kit (Beyotime, China). The mixture was placed in a dark environment at room temperature. The mixture was incubated at room temperature in a dark environment for 10-20 minutes, after which flow cytometry was performed to detect the apoptosis rate.

EdU incorporation assay

Cells were inoculated on a Nunc™ Lab-Tek™ II Chamber Slide™ System (#154453, Thermo Scientific) and after waiting for cells to attach to the wall, they were incubated for 2 hours in a medium containing 10 μM EdU. Cell proliferation rate was assayed using BeyoClick™ EdU Cell Proliferation Kit with Alexa Fluor 594 (Reyotime, China) according to the instructions, after which cells were observed by fluorescence

microscopy. The proliferation rate was calculated by the ratio of EdU-positive nuclei to total nuclei.

Transwell assay to determine cell migration

Serum-free medium was added to the top chamber of the transwell (Corning, USA) and a standard medium containing 20% FBS medium was added to the bottom chamber. Afterward, 5 × 10^4 cells were inoculated in the top chamber of the transwell (pore size of 8.0 μm). After 24-36 hours of incubation, the cells were fixed with 90% anhydrous ethanol for 30 minutes, stained with 0.1% crystal violet (Solarbio Technology, China) for 30 minutes, washed, and photographed in five randomly selected fields under the microscope.

Xenograft mouse experiments and in vivo studies

All animal procedures were approved by the Animal Ethical and Welfare Committee of the Academy of Military Medical Sciences (Ethics Committee approval IACUC-DWZX-2023-052). The mice used in the study were 4-week-old female nude mice housed in a laminar flow air unit under sterile conditions with a 12-hour light/12-hour dark cycle and fed ad libitum. LIM-R cells expressing luciferase (3×10^6 cells/mouse) were injected subcutaneously into the right side of nude mice. Tumors were established for 15 days and then vernier caliper measurements were taken of subcutaneous tumors

in mice to obtain pre-treatment tumour volumes. This was followed by treatment with intraperitoneal injection of cetuximab (1 mg/mouse/3 days) and intragastric administration of celecoxib (10 mg/kg/3 days). The size of the insitu tumors was monitored by the bioluminescence channel of the IVIS Spectrum every 4-6 days during the period. mice were euthanized after 30 days.

Statistical analyses

GraphPad Prism 8 and R version 4.3.0 were used for statistical analysis. Data were collected based on at least 3 or more experiments and expressed as mean ± standard deviation. Differences between groups were compared by independent samples t-test or two-way ANOVA. The *p*-value < 0.05 indicates statistically significant differences.

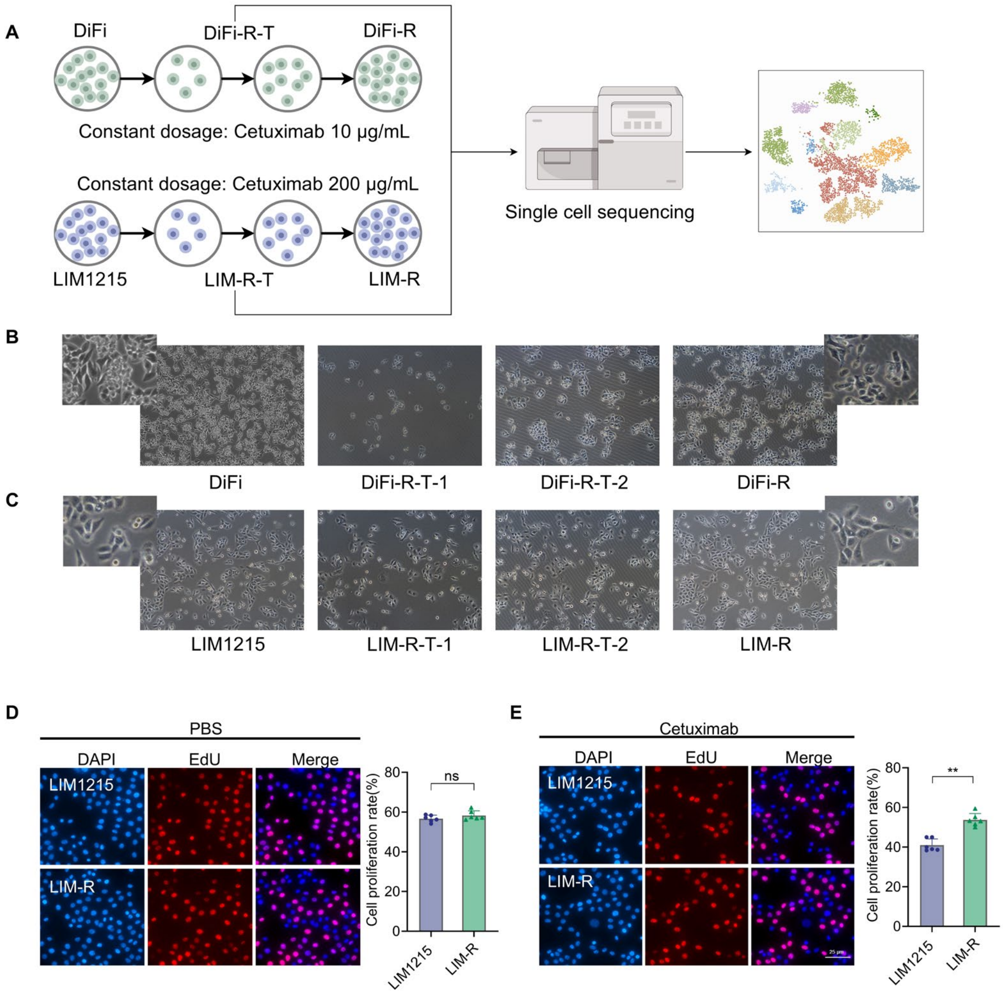

Figure S1 Establishment of cetuximab resistance model.

(A) Flow of Cetuximab resistance cell model establishment. (B) Morphological observation of DiFi cell line during cetuximab-induced resistance. (C) Morphological observation of LIM1215 cell line during cetuximab-induced resistance. (D) EdU assay to detect the proliferation rate of the resistant line LIM-R versus the sensitive parental line LIM1215 when cetuximab was not present. (E) EdU assay to detect the proliferation rate of the resistant line LIM-R versus the sensitive parental line LIM1215 when cetuximab is present.

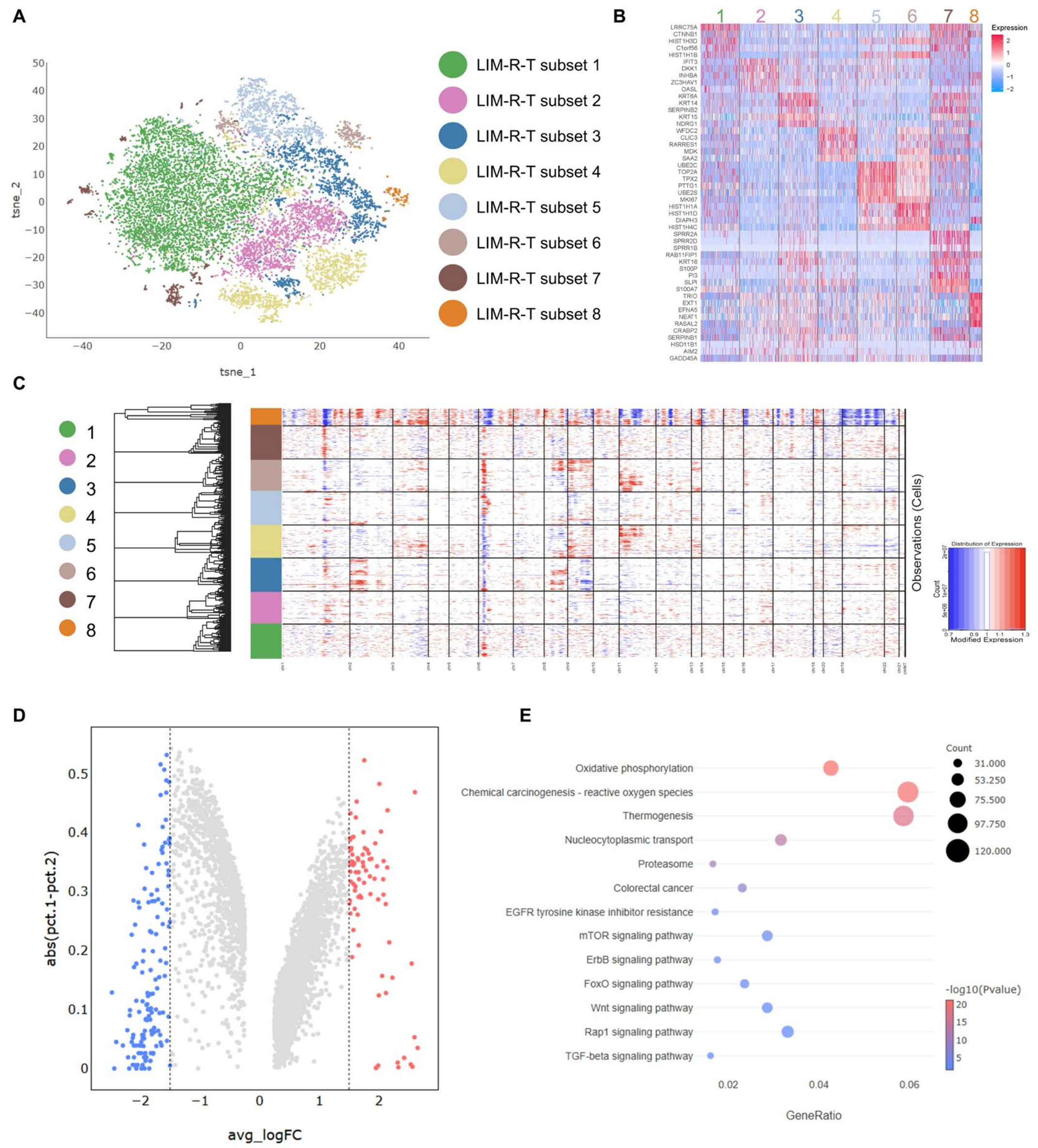

Figure S2 Single-cell sequencing atlas of the drug resistance transition cell line LIM-R-T.

(A) t-SNE visualization of multiple cell subsets within LIM-R-T. (B) Heatmap of Marker genes characteristically highly expressed in each cell subset. (C) Inferred copy number variation (inferCNV) of each cell subset. (D) Volcano plot visualization of Differentially expressed genes (DEGs) derived by comparing subset 2 with other subsets. (E) KEGG analysis of DEGs.

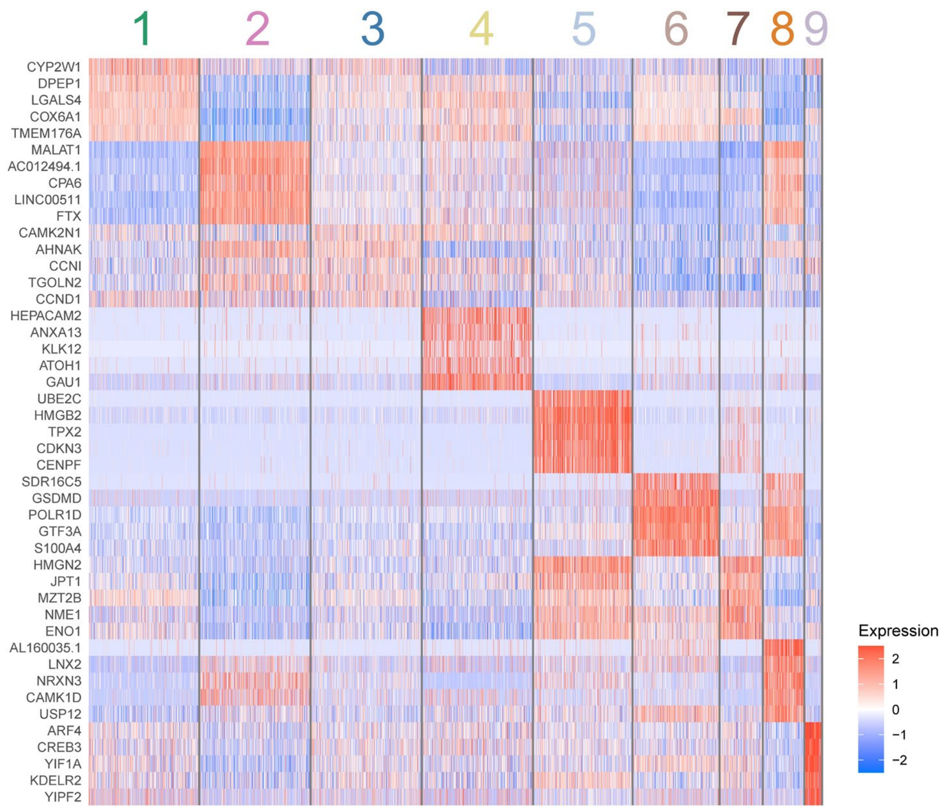

Figure S3 Heatmap of Marker genes characteristically highly expressed in each cell subset within DiFi-R-T.

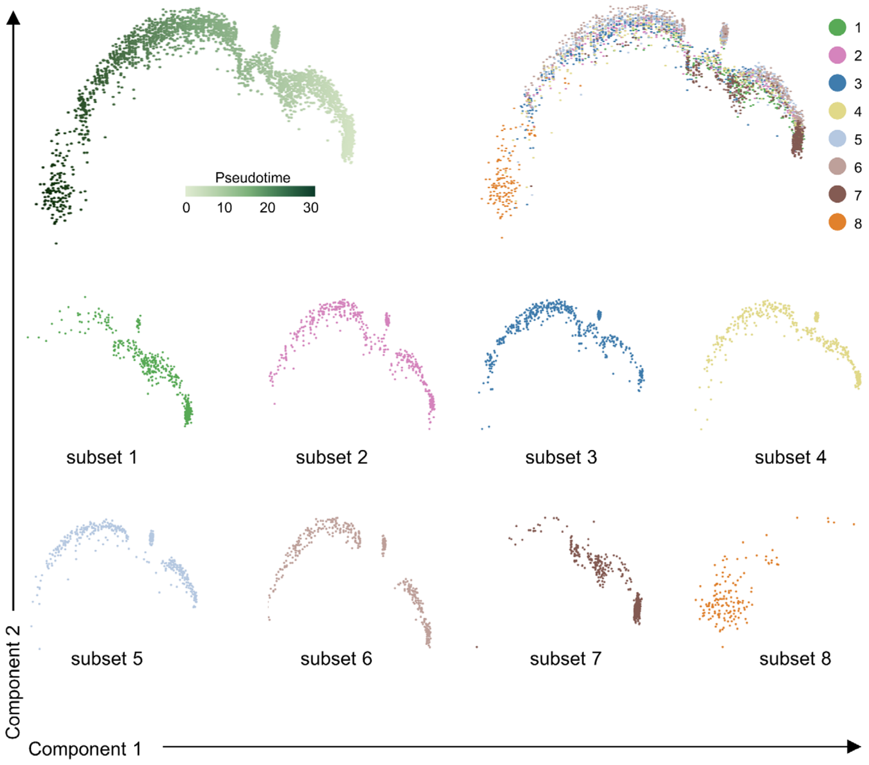

Figure S4 Distribution of the various cell subsets of LIM-R-T on the pseudotime line.

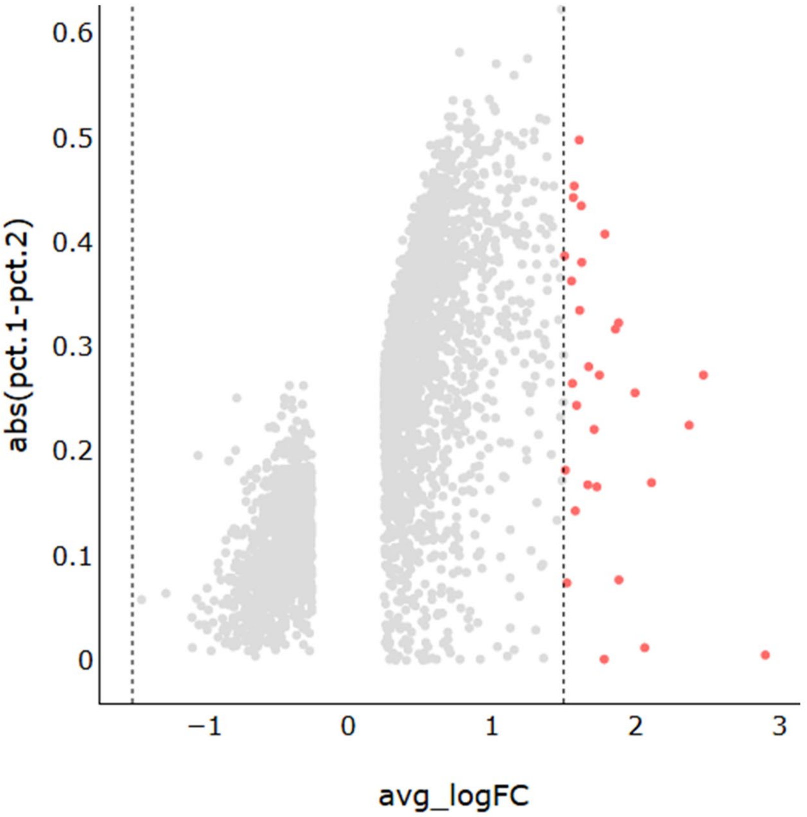

Figure S5 Volcano plot visualization of Differentially expressed genes (DEGs) derived by comparing subset 2 with other subsets.

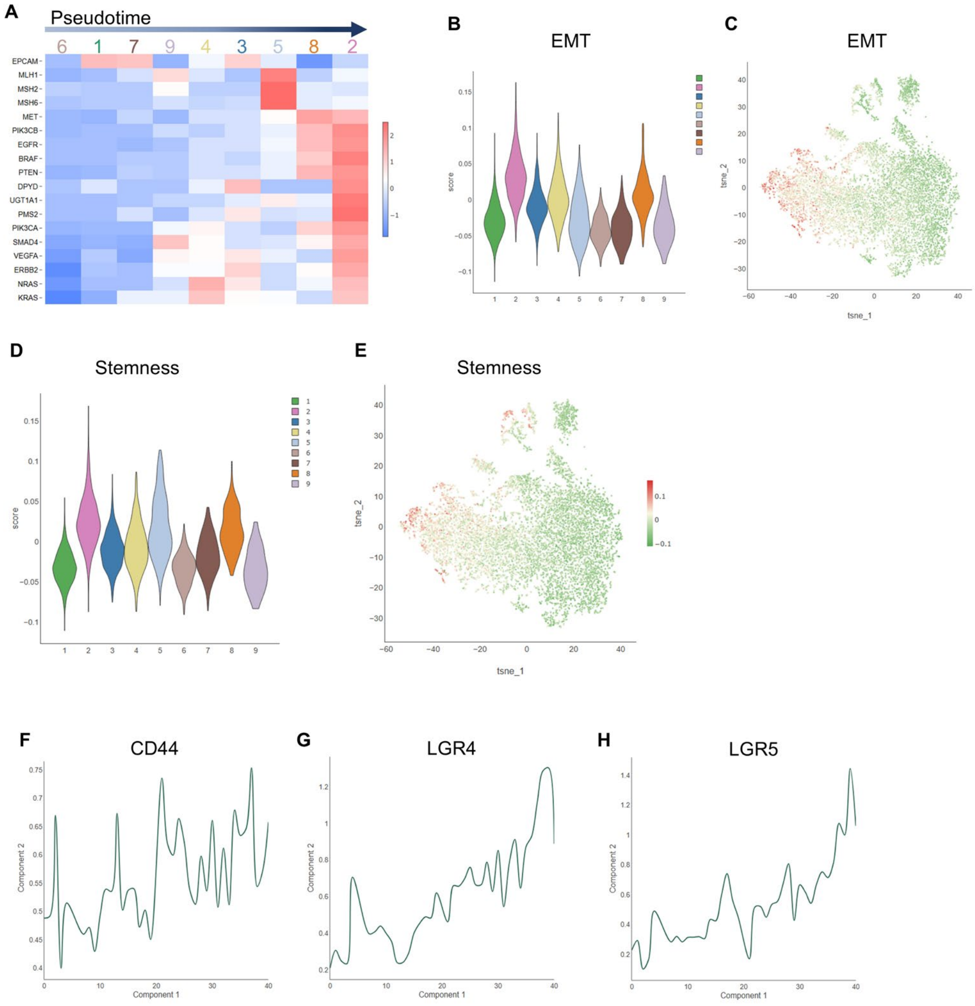

Figure S6 Following pseudotime, multiple genes associated with colorectal cancer treatment, tumor stemness and EMT were progressively up-regulated in DiFi-R-T.

(A) Following pseudotime, heatmap of colorectal cancer treatment-related gene expression. (B) Violin plots of EMT gene set expression scores for each cell subset. (C) Projection of EMT gene set expression on the t-SNE plot. (D) Violin plots of tumor stemness gene set expression scores for each cell subset. (E) Projection of tumor stemness gene set expression on the t-SNE plot. (F) Characteristic line plot of tumor stemness-related gene-CD44 expression changes following pseudotime. (G)

Characteristic line plot of tumor stemness-related gene-LGR4 expression changes following pseudotime. (H) Characteristic line plot of tumor stemness-related gene-LGR5 expression changes following pseudotime.

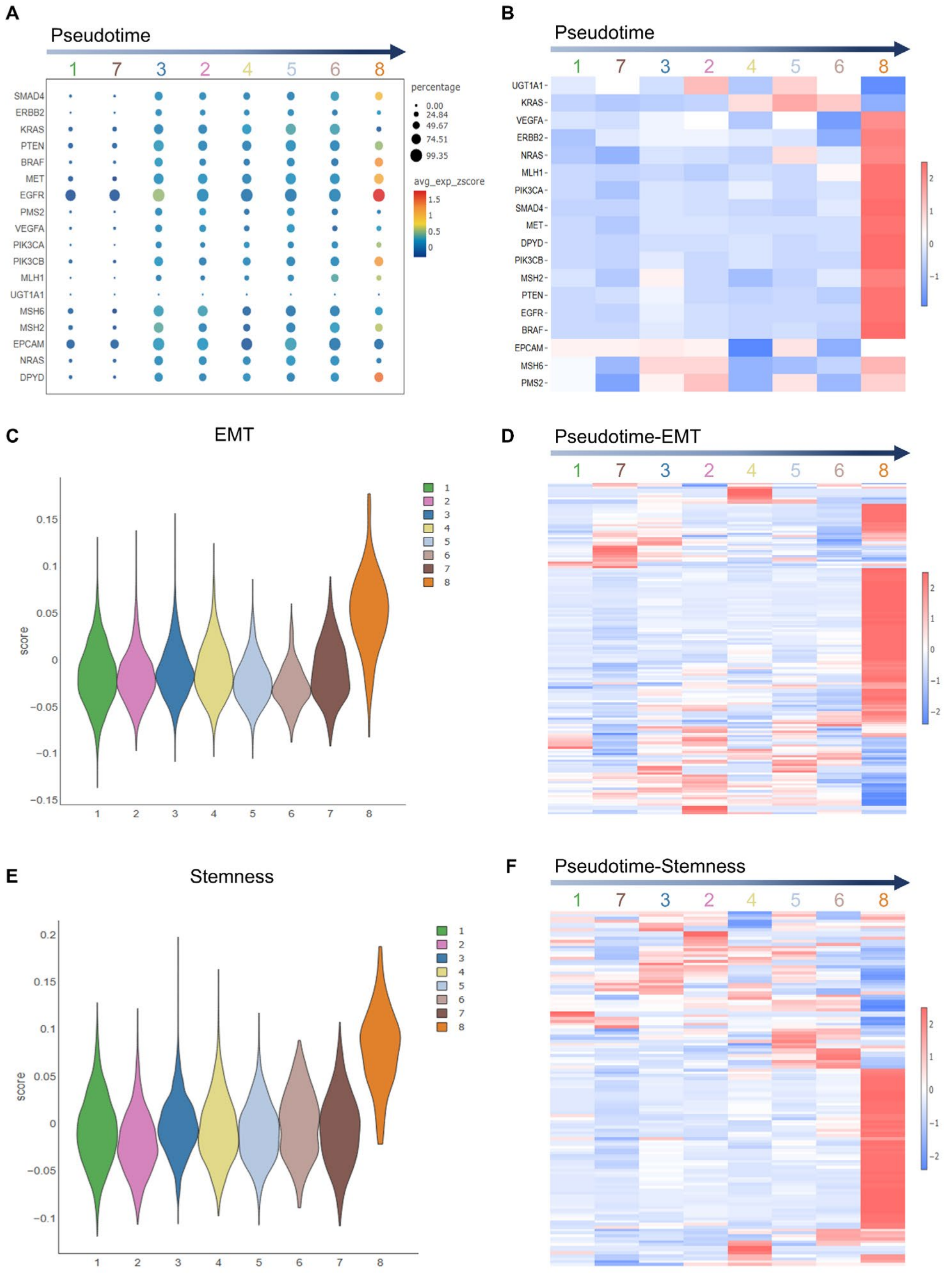

Figure S7 Following pseudotime, multiple genes associated with colorectal cancer treatment, tumor stemness and EMT were progressively up-regulated in LIM-R-T.

(A) Bubble plots of colorectal cancer treatment-related gene expression following pseudotime. (B) Following pseudotime, heatmap of colorectal cancer treatment-related gene expression.(C) Violin plots of EMT gene set expression scores for each cell subset.

(D) Heat map of EMT gene set expression. (E) Violin plots of tumor stemness gene set expression scores for each cell subset. (F) Heat map of the expression of the tumor stemness gene set.

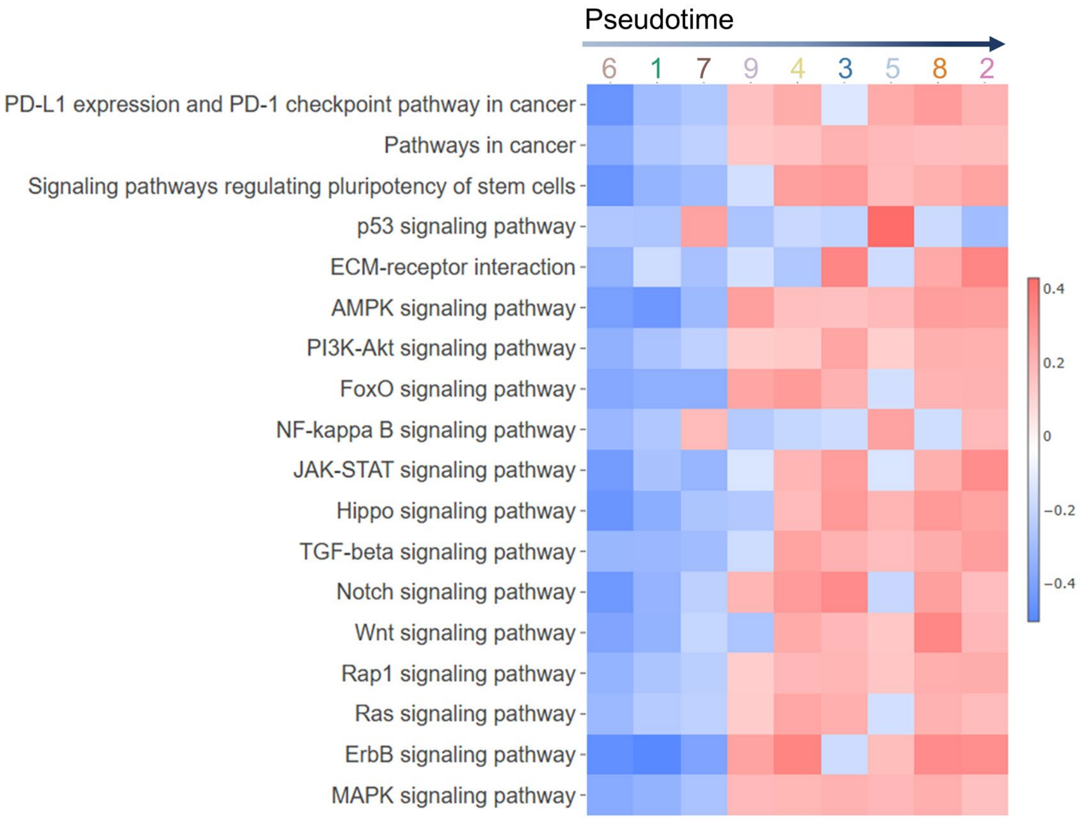

Figure S8 GSVA analysis reveals changes in multiple tumor-related signaling pathways following pseudotime in DiFi-R-T.

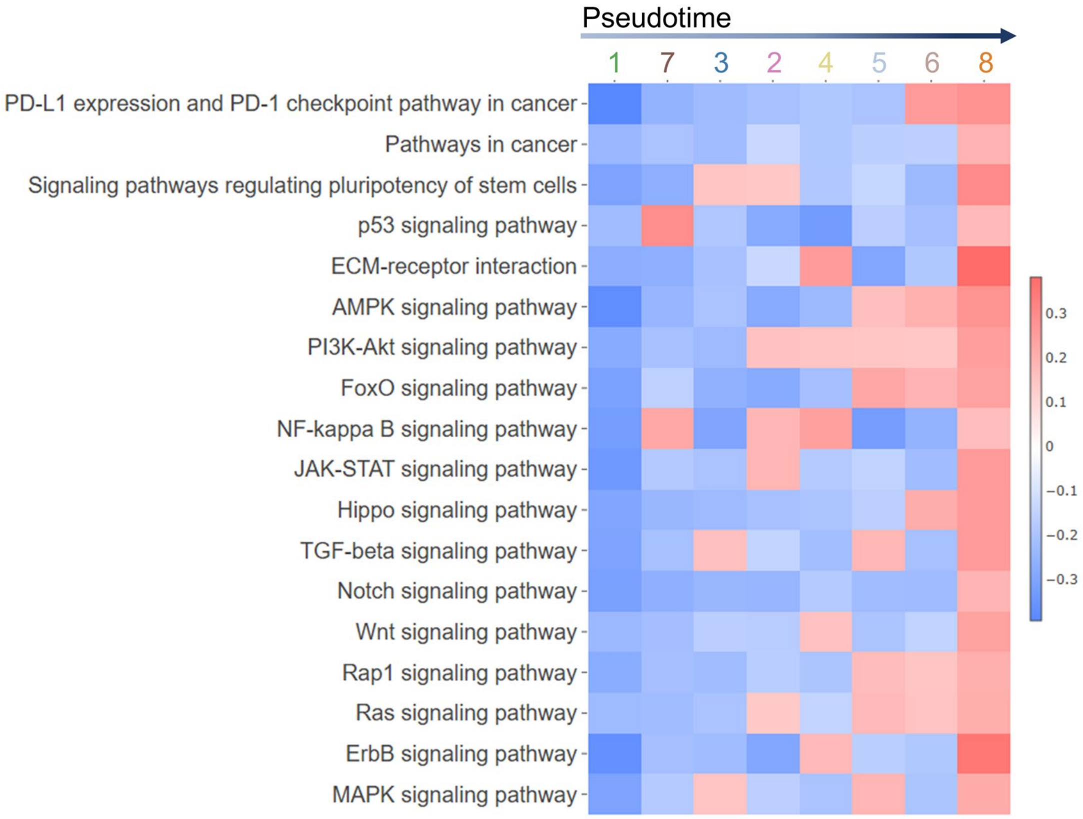

Figure S9 GSVA analysis reveals changes in multiple tumor-related signaling pathways following pseudotime in LIM-R-T.

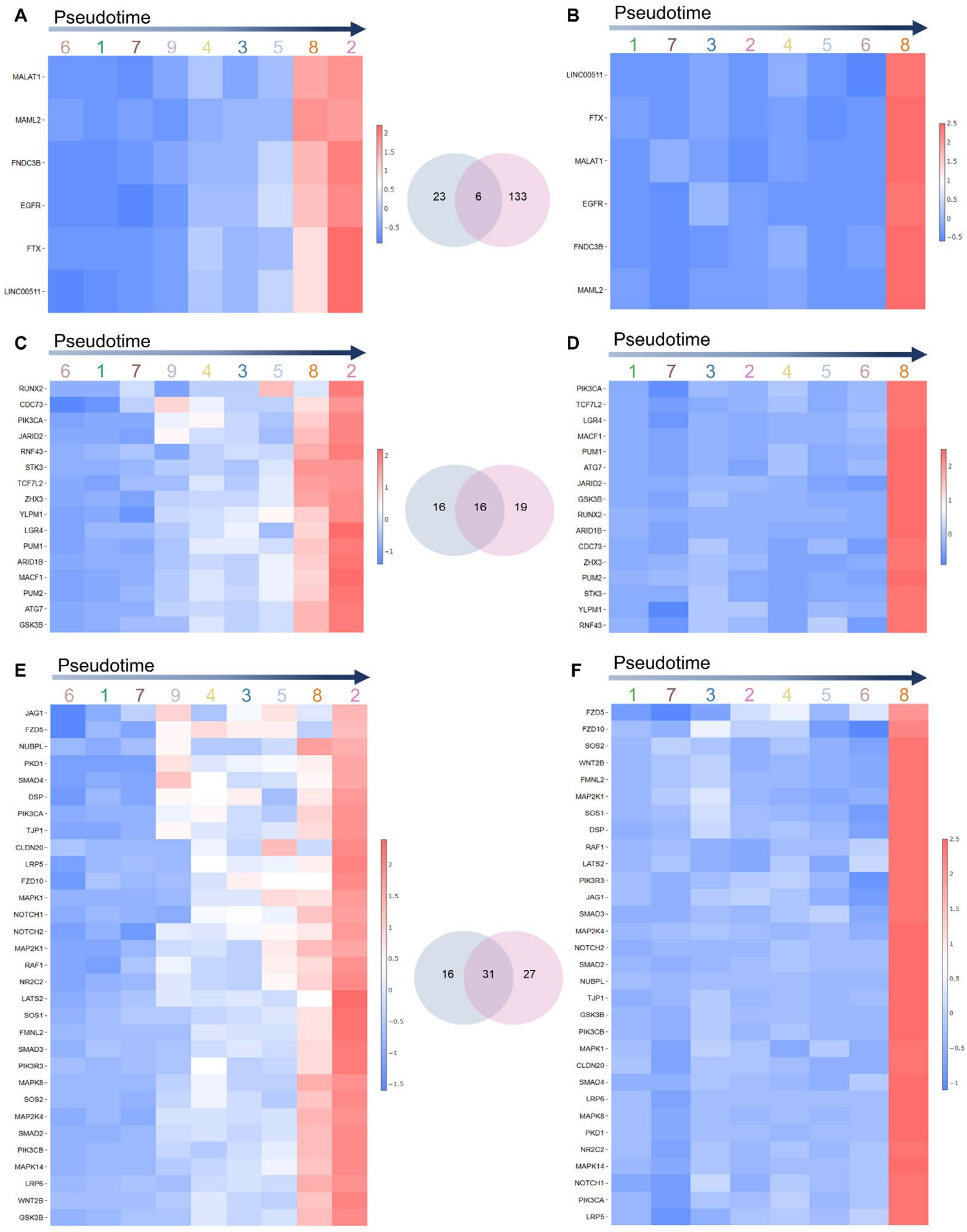

Figure S10 Evolutionary trends in key intersecting genes derived from DiFi-R-T and LIM-R-T single-cell sequencing analyses following pseudotime.

(A) Evolutionary trends of intersecting genes derived from comparison of endpoint resistance clusters with other subgroups in DiFi-R-T following pseudotime.(B) Evolutionary trends of intersecting genes derived from comparison of endpoint

resistance clusters with other subgroups in LIM-R-T following pseudotime. (C) Evolutionary trends of stemness intersection genes in DiFi-R-T following pseudotime. (D) Evolutionary trends of stemness intersection genes in LIM-R-T following pseudotime. (E) Evolutionary trends of EMT intersection genes in DiFi-R-T following pseudotime. (F) Evolutionary trends of EMT intersection genes in LIM-R-T following pseudotime.

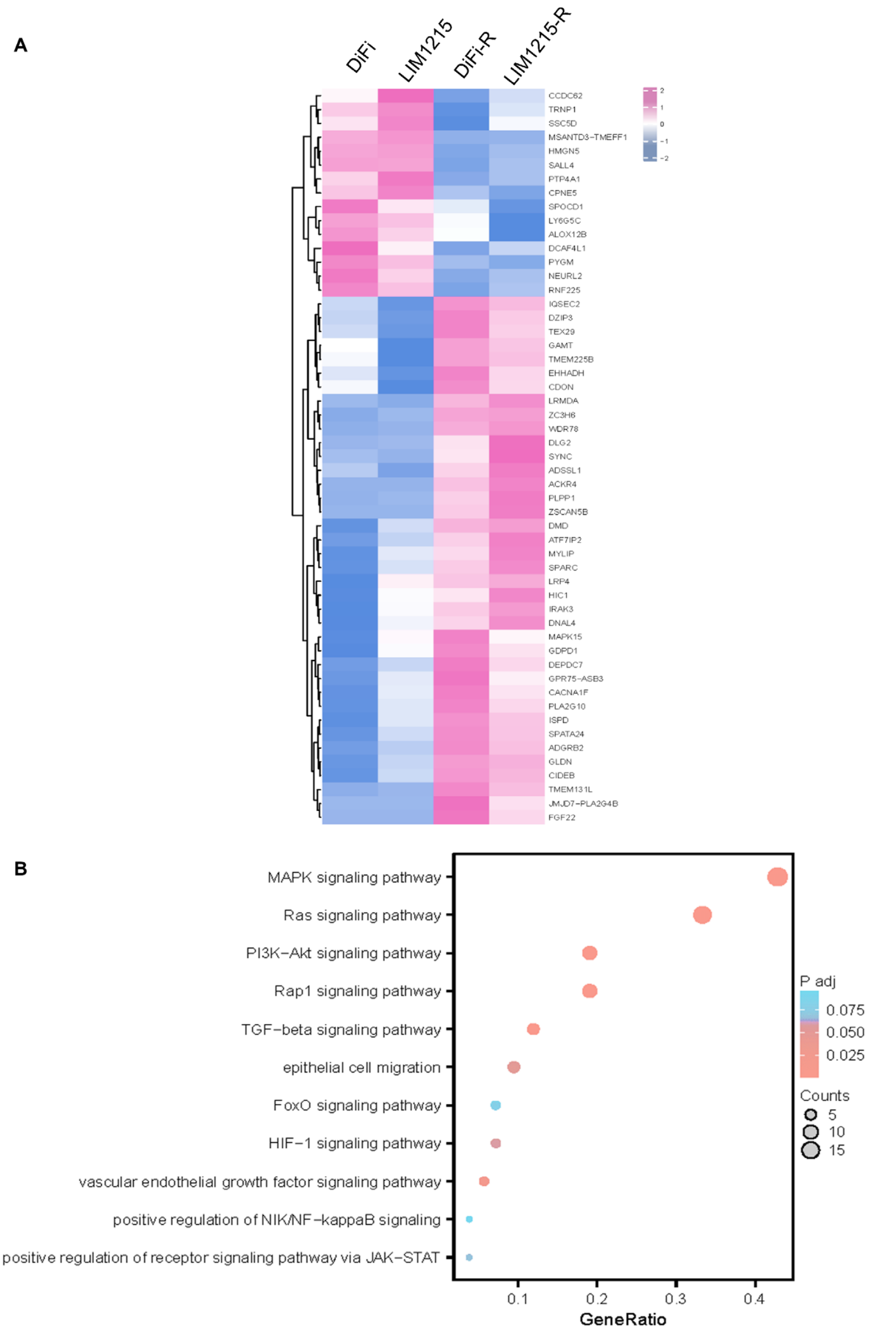

Figure S11 RNA-seq analysis of intestinal cancer cell lines before and after cetuximab resistance.

(A) Analysis of DEGs before and after cetuximab resistance. (B) KEGG analysis of DEGs.

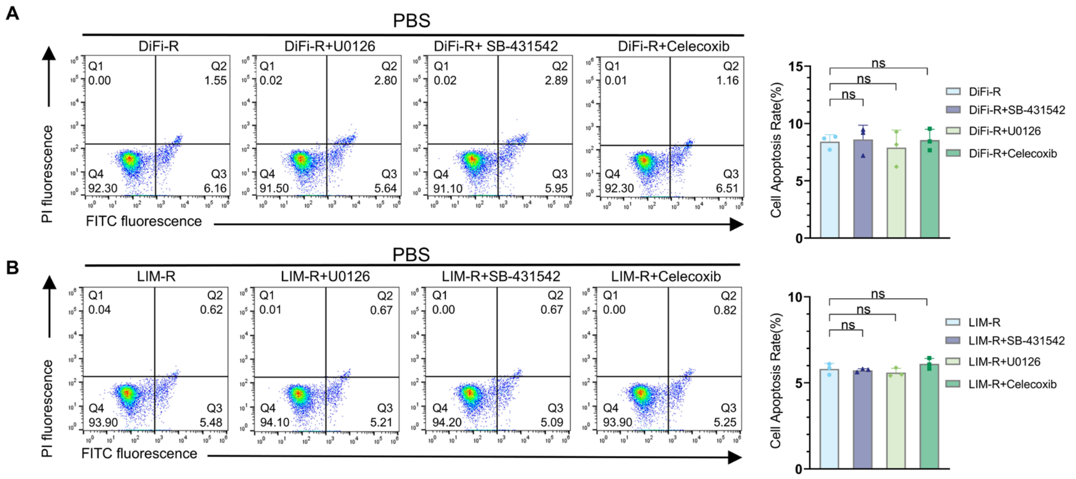

Figure S12 Apoptosis rates of the parental sensitive line, the control group of the resistant line, the U0126 group, the SB-431542 group and the celecoxib group when cetuximab was not present.

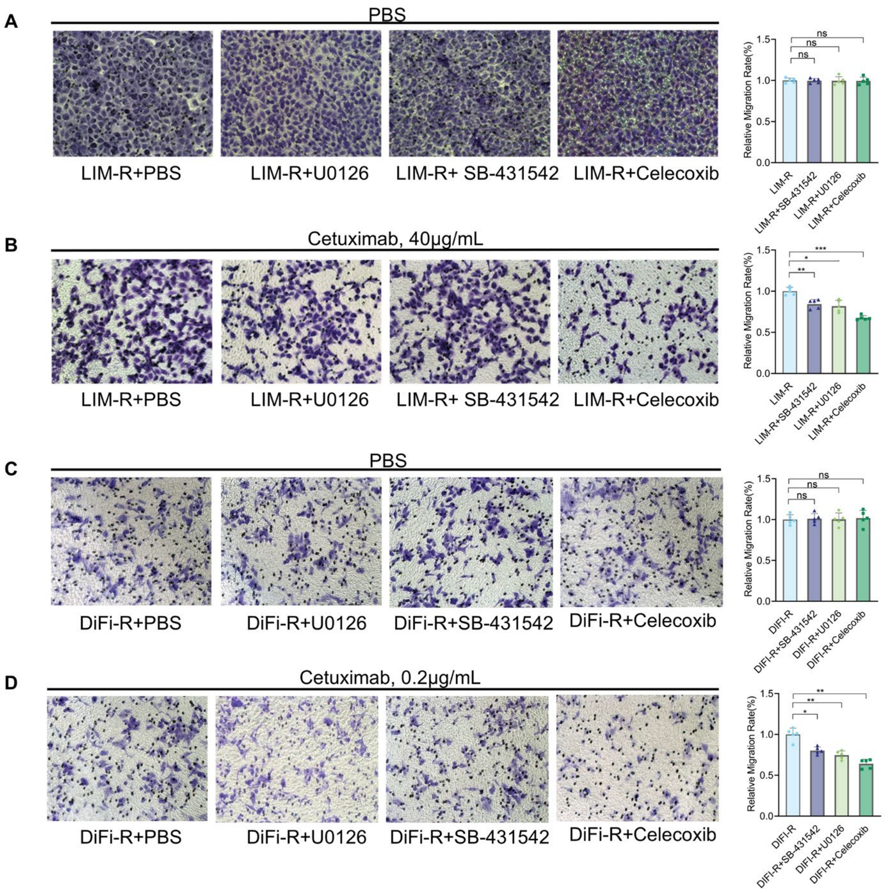

Figure S13 Celecoxib can synergistically inhibit the migration of drug-resistant lines when combined with cetuximab.

(A) Transwell assay migration rate of LIM-R in each group when not combine d with cetuximab. (B) Transwell assay for the migration rate of LIM-R in cont rol, celecoxib, SB-431542 and U0126 groups when combined with cetuximab (40 μg/mL). (C) Transwell assay for the migration rate of DiFi-R in each grou p when not combined with cetuximab. (D) Transwell assay for the migration r ate of DiFi-R in control, celecoxib, SB-431542 and U0126 groups when combi ned with cetuximab (0.2 μg/mL). Data are expressed in mean ± SEM; *P<0.05, **P<0.01,***P<0.001.*P<0.05,**P<0.01,***P<0.001,****P<0.0001.

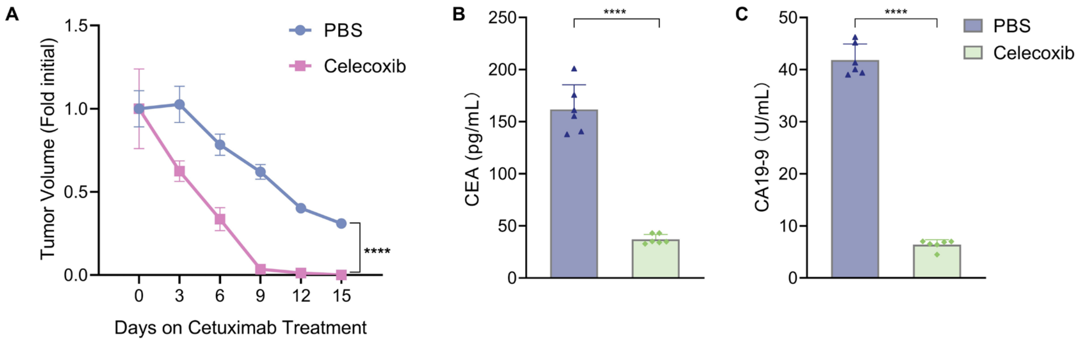

Figure S14 The combination of celecoxib and cetuximab synergistically inhibits CRC growth in vivo.

(A) Change curves of subcutaneous tumor volume in nude mice in the PBS group and celecoxib group during cetuximab treatment. (B) CEA levels in plasma of nude mice in the PBS group and celecoxib group at the endpoint of cetuximab treatment. (C) Plasma levels of CA19-9 in nude mice in the PBS and celecoxib groups at the endpoint of cetuximab treatment.
